# Perioperative Blood Management on a Successful Surgical Operation of a Patient With Congenital Coagulation Factor V Deficiency

**DOI:** 10.1002/ccr3.70888

**Published:** 2025-09-09

**Authors:** Ronghui Shi, Jianjun Wu, Qiang Meng, Ji Nie, Li Zhang, Xiaoqiong Long, Lan Yang, Shuming Zhao

**Affiliations:** ^1^ Department of Blood Transfusion Guiqian International General Hospital Guiyang Guizhou China; ^2^ Department of Clinical Laboratory Guiqian International General Hospital Guiyang Guizhou China; ^3^ Precision Medicine Center Guiqian International General Hospital Guiyang Guizhou China

**Keywords:** coagulation factor V deficiency, cryoprecipitated antihemophilic factor, fresh frozen plasma (FFP), thrombelastogram (TEG)

## Abstract

Thrombelastogram may provide a more accurate assessment of coagulation condition and transfusion strategy than the traditional coagulation profile and factor assays, and may be a useful ancillary tool guiding treatment decisions in factor V deficiency patients undergoing surgical procedures.

## 1 Introduction

Factor V (FV) deficiency, inherited or acquired, is a rare bleeding disease with an estimated prevalence of 1 per 1 million live births [1, 2]. Patients with FV deficiency may be bleeding externally or internally, from mild mucosal bleeding to severe life‐threatening hemorrhage. Patients usually need to improve their FV levels to more than 25%–30% by infusion of fresh frozen plasma (FFP) or cryoprecipitated antihemophilic factor (cryo) to ensure hemostasis and surgical safety during bleeding episodes or operative procedures. Platelets can be transfused when patients with FV deficiency have severe bleeding and do not respond well to conventional treatment [2]. The coagulation profile and factor assays are usually used to assess the coagulation status. In recent years, more and more scholars have applied global coagulation assays (such as Thromboelastogram, TEG; or thrombin generation, TG) for the clinical transfusion evaluation of surgical patients [3]. These global coagulation assays can provide information about the coagulation and fibrinolysis phases of the coagulation process, which includes the interactions of various blood components (platelets, plasma, and leukocytes) to guide transfusion therapy during surgeries. In this report, we present a patient with FV deficiency who underwent a surgical procedure. We used the abovementioned methods to assess and manage blood transfusion strategies.

## 2 Case Presentation/Examination

A 31‐year‐old male patient (weight 60 kg) presenting with recurrent upper abdominal pain for over 2 years was admitted to the gastroenterology surgery department. His medical record indicated that the patient had been diagnosed with suspected FV deficiency without a genetic positive result prior to admission, but had not exhibited symptoms of bleeding. He had previously undergone head debridement 1 year ago in another hospital by compressing the wound to stop the bleeding.

## 3 Methods (Differential Diagnosis, Investigations, and Treatment)

**FIGURE 1:**
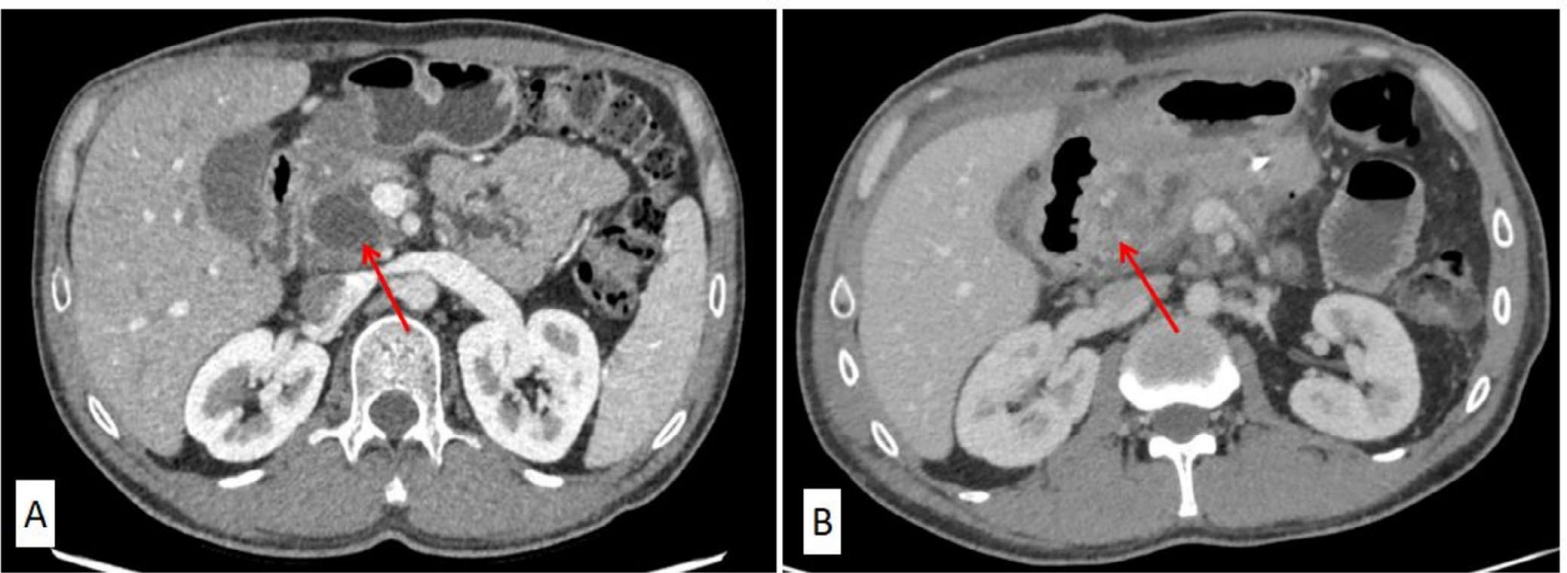
CT scan of pancreatic and intestinal snout area before (A) and after (B) pancreatic cyst surgery.

Upon admission, the blood sample was tested for coagulation profile, factor assay, and TEG. The coagulation profiles indicated prolonged prothrombin time (PT) (22.40 s; normal range of 11–14.5 s) and activated partial thromboplastin time (aPTT) (63.20 s; normal range of 28–44 s). Factor assays performed twice confirmed that the FV activity was only 1.9% and 1.8%, respectively (reference range, 102.4% ± 30.9%). The other factors, II, VII, VIII, IX, X, XI, and XII, did not change significantly. TEG parameters were normal with an *R*‐value of 7.2 (*R*‐time, reaction time, normal range of 5–10 min), *K* value of 2.3 (*K*‐time, normal range of 1–3), *α*‐angle of 59.1 (normal range of 53–72), MA value of 65.0 (maximum amplitude, normal range of 50–70), and lysis at 30 min of 0 (LY30, 0%–75%). An abdominal CT scan indicated a potential pancreatic head pseudocyst (Figure 1A). The patient was finally diagnosed with pancreatic pseudocyst, pancreatic calcification in the head region, and FV deficiency. After signing a patient written informed consent statement, the patient was then prepared for blood transfusion and operation.

The patient was infused with blood components to correct the coagulation function and then underwent surgery by monitoring coagulation status with a coagulation profile, factor assay, and TEG. In order to evaluate the coagulation function and tolerance, and assess the safety and feasibility of surgery, the patient was alternately administered FFP (1200 mL, 20 mL/kg) and cryo (10 U) infusions after 3 days of rest. For post‐transfusion evaluation purposes, these tests were performed before transfusion as well as at 1, 24, and 48 h post‐infusion. Meanwhile, a blood sample was also sent to detect gene mutation by molecular genetics [4].

The patient's PT and aPTT were initially prolonged to 23.3 and 62.1 s, respectively. After transfusion of 1200 mL of FFP, PT and aPTT before and after 1, 24, and 48 h of infusion were 23.3, 20.1, 21.4, and 22.4 s, and 62.1, 54.5, 54.4, and 61.4 s, respectively. After infusion of 10 U of cryo, the PT and aPTT before and after 1, 24, and 48 h of infusion were 22.6, 15.7, 19.4, and 20.8 s, and 59.1, 43.4, 53.6, and 54.4 s, respectively. The FV activity after 24 h of infusion of FFP and cryo increased from 1.9% and 1.8% to 5.1% and 6.0%, respectively. The changes in other factors were not significant. The TEG parameters on and after 1, 24, and 48 h of infusion of FFP or cryo were all within normal ranges.

**FIGURE 2:**
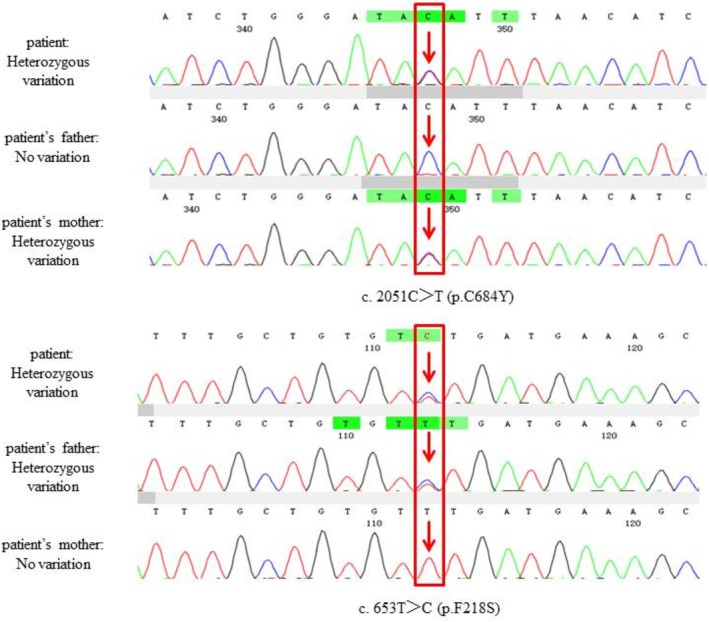
Molecular analyses of the *F5* gene in the patient with congenital factor V deficiency. Variant validation may result in reference sequence complementation, for example, G>A can also be expressed as C>T. The genetic variant analysis of the blood system hereditary disease gene panel was conducted by NGS on the exonic regions and adjacent splice sites of the genes included in the panel. The following BAM (Binary Alignment Map) figure shows that the tested patient carries two heterozygous mutations in the *F5* gene: upper portion: F5: NM_000130.5: exon 13: c.2051G>A: p.C684Y and lower portion: F5: NM_000130.5: exon 5: c.653T>C: p.F218S. Sanger sequencing was used to validate the next‐generation sequencing results, and parental testing was performed on both variants. Variant 1 (upper portion) was inherited from the patient's mother, and the patient's father did not show the related variant. Variant 2 (lower portion) was inherited from the patient's father, and the patient's mother did not show the related variant. Variants 1 and 2 together form a compound heterozygous mutation. (The images from top to bottom show the samples of the patient, the patient's father, and the patient's mother.)

The patient's genetic test revealed two heterozygous mutations in the *F5* gene, and the heterozygous variants were confirmed. Genomic DNA was extracted from peripheral blood leukocytes. The *F5* exon adjacent splicing region (about 20 bp) was captured by probe hybridization, enriched, and sequenced by next‐generation sequencing (NGS). As a result, two different heterozygous mutations in *F5* were identified. The red arrows indicate the mutated nucleotides. Variant c.2051G>A was found in his mother, and variant c.653T>C was found in his father, as illustrated in Figure 2. Both variants were likely pathogenic according to the American College of Medical Genetics and Genomics (ACMG) and the Association for Molecular Pathology (AMP) criteria.

Considering the TEG parameters within normal ranges, the surgery was performed after prophylactic infusion of 700 mL of FFP, fluid restriction, management of airway pressure, and controlling bleeding by central venous pressure reduction (Figure 1B). The operative time was 7 h, and the amount of intraoperative bleeding loss was 220 mL. The patient was then prophylactically transfused 500 mL of FFP at 2 h after the surgery, and subsequently 250–500 mL of FFP every day for 1–7 days to improve coagulation status.

## 4 Conclusion and Results

The patient underwent surgery after receiving appropriate FFP transfusion. Bleeding was minimized during the operation and no bleeding tendency was observed. Up to 9 days after surgery, PT and aPTT were prolonged slightly, TEG parameters remained within the normal ranges, and hemoglobin (HGB) level was 122–110 g/L and did not change significantly. The patient had no significant postoperative wound bleeding due to our blood transfusion strategy and was discharged on postoperative day 12.

## 5 Discussion

Factor V deficiency is a rare bleeding disorder and may be regarded as mild, moderate, or severe stage based on FV activity levels. In mild deficiency, the FV level exceeds 10% of the normal activity level in plasma. In moderate and severe stage, FV levels are 1% to 10% and less than 1%, respectively [5]. Approximately 80% of FV circulates in plasma, and 20% is stored in platelet α‐granules [2]. In humans, plasma FV is produced by hepatocytes and platelet FV is originated from plasma FV via endocytosis by megakaryocytes. The plasma level of FV is 7 μg/mL. The factor has a half‐life of 20 h on average (between 12 and 36 h). The clinical manifestations of FV deficiency primarily involve visceral and mucosal bleeding, with joint and muscle involvement being uncommon. The disease manifestation is similar to other coagulation factor deficiencies, with bleeding symptoms ranging from minor to severe and life‐threatening levels [5].

Initial laboratory values in a patient with FV deficiency show prolonged PT and aPTT. A low plasma FV level can validate the diagnosis. Molecular genetic analysis is usually used to sequence the *F5* gene, placed on chromosome 1q24.2, to identify mutations to diagnose inherited FV deficiency [4, 5, 6, 7]. Congenital FV deficiency, although rare, can cause uncontrolled bleeding. Acquired FV deficiency, which arises from FV inhibitor or drug‐induced production, is less common than the inherited form [8, 9]. Diagnosis and evaluation of the hemodynamic status of patients with FV deficiency usually require a coagulation profile and blood tests to measure the level and activity of various coagulation factors, including FV [5]. For the congenital form, getting an accurate family history of bleeding status may be the key step to early diagnosis. All factor activity levels should be tested to verify a coexisting deficiency, as in combined factors V and VIII deficiency.

In recent years, viscoelastic assays such as TEG have shown the ability to guide the rational use of blood and its components, and the hemostatic recovery of injured bleeding patients [10, 11]. These tests can indicate patients' coagulation status, and when compared with other available parameters such as hemoglobin and blood gas, can provide assistance with early patient blood management by the clinician. TEG is also routinely used to guide transfusion protocols in surgical patients, compared to conventional coagulation assays (i.e., PT, aPTT, fibrinogen level, platelet count) to reduce the use of plasma and platelet blood products. Normal TEG parameters can even lead us to perform surgery without any blood component support [3].

Improving the patient's coagulation function depends on the patient's etiology and severity [5, 10, 11]. Mild to severe inherited FV deficiency may be managed with the infusion of FFP and cryo to replenish FV levels. Perioperative blood management in the patient with congenital FV deficiency undergoing surgery requires pre‐transfusion and post‐transfusion evaluation in combination with laboratory tests and clinical manifestations [6, 7]. The safety of surgical operation is unclear as there are few available reports [11, 12]. Considering the patient's abnormal coagulation PT and aPTT, a comprehensive hospital consultation was conducted. Some clinicians stated that patients with FV deficiency were theoretically contraindicated from surgery. Surgery can be performed if the coagulation factor activity improves to at least 15%–20% [1]. The coagulation tests, factor assays, and TEG were assayed and showed that PT and aPTT were slightly reduced, and TEG parameters were normal in the patient after the transfusion. These results indicated that the coagulation status had been partially corrected, although it was difficult to reach the FV activity of 25% to 30% which is considered the safe level for surgery. Therefore, we used prophylactic transfusions of a certain amount of FFP to maintain coagulation and prevent bleeding before and after the surgery.

Our case has previously been diagnosed with suspected FV deficiency. In order to ensure the safety of the surgery and avoid the risk of heavy bleeding, a certain amount of FFP should be transfused to maintain the stability of coagulation function. To evaluate the true coagulation function and FV activity in this patient, we adopted the method of alternating transfusion of FFP and cryo at the basal level before the surgery. The results showed that FV activity could be slightly increased, though it was far from the safe level for surgery reported in the literature. The surgery with only FFP infusion and no tranexamic acid used was undergone smoothly for 7 h without any noticeable intraoperative bleeding. These results suggest that TEG may provide a more accurate assessment of coagulation condition and transfusion strategy than the traditional coagulation profile and factor assays in judging the clotting status of patients with FV deficiency. TEG may be a useful ancillary tool guiding treatment decisions in FV deficiency patients undergoing surgical procedures.

## Author Contributions

**Ronghui Shi:** conceptualization, data curation, project administration, resources, writing – original draft. **Jianjun Wu:** conceptualization, data curation, writing – original draft, writing – review and editing. **Ji Nie:** formal analysis, methodology. **Li Zhang:** methodology. **Qiang Meng:** methodology. **Xiaoqiong Long:** methodology. **Lan Yang:** methodology. **Shuming Zhao:** supervision.

## Consent

The patient's written informed consent form is in Chinese, which basically means, “I, Luo Shi, have known and understood that I am being treated in this hospital, and the doctor has informed me that there will be some risks during the blood transfusion and surgery, and I am aware and willing to bear the risks. I agreed and signed the document and also I agreed to have my case with images published in a medical journal.”

## Conflicts of Interest

The authors declare no conflicts of interest.

## Data Availability

The data that support the findings of this study are not openly available due to reasons of patient personal and hospital privacy and are available from the corresponding author upon reasonable request. Data are located in controlled access data storage at Guiqian International General Hospital.
